# Charter to establish clinical exercise physiology as a recognised allied health profession in the UK: a call to action

**DOI:** 10.1136/bmjsem-2021-001158

**Published:** 2021-09-21

**Authors:** Helen Jones, Keith P George, Andrew Scott, John P Buckley, Paula M Watson, David L Oxborough, Dick H Thijssen, Lee E F Graves, Greg P Whyte, Gordon McGregor, Louise H Naylor, Michael Rosenberg, Christopher D Askew, Daniel J Green

**Affiliations:** 1 Research Institute for Sport and Exercise Sciences, Liverpool John Moores University, Liverpool, UK; 2 School of Sport, Health and Exercise Science, University of Portsmouth, Portsmouth, UK; 3 Centre for Active Living, University Centre Shrewsbury, University of Chester, Shrewsbury, UK; 4 Radboud Institute for Health Sciences, Radboud University Medical Centre, Radboud, The Netherlands; 5 University of Coventry, Coventry, UK; 6 University Hospitals Coventry and Warwickshire NHS Trust, Coventry, UK; 7 School of Human Sciences (Exercise and Sport Sciences), The University of Western Australia, Perth, Western Australia, Australia; 8 School of Health and Behavioural Sciences, University of the Sunshine Coast, Sunshine Coast, Queensland, Australia; 9 Sunshine Coast Health Institute, Sunshine Coast Hospital and Health Service, Sunshine Coast, Queensland, Australia

**Keywords:** exercise, exercise physiology, exercise rehabilitation, rehabilitation

## Abstract

The UK population is growing, ageing and becoming increasingly inactive and unfit. Personalised and targeted exercise interventions are beneficial for ageing and the management of chronic and complex conditions. Increasing the uptake of effective exercise and physical activity (PA) interventions is vital to support a healthier society and decrease healthcare costs. Current strategies for exercise and PA at a population level mostly involve self-directed exercise pathways, delivered largely via the fitness industry. Even for those who opt-in and manage to achieve the current recommendations regarding minimum PA, this generic ‘one-size-fits-all’ approach often fails to demonstrate meaningful physiological and health benefits. Personalised exercise prescription and appropriate exercise testing, monitoring and progression of interventions for individuals with chronic disease should be provided by appropriately trained and recognised exercise healthcare professionals, educated in the cognate disciplines of exercise science (eg, physiology, biomechanics, motor control, psychology). This workforce has operated for >20 years in the Australian public and private healthcare systems. Accredited exercise physiologists (AEPs) are recognised allied health professionals, with demonstrable health and economic benefits. AEPs have knowledge of the risks and benefits of distinct forms of exercise, skills in the personalised prescription and optimal delivery of exercise, and competencies to support sustained PA behavioural change, based on the established scientific evidence. In this charter, we propose a road map for the training, accreditation and promotion of a clinical exercise physiology profession in the UK.

**Key points**

- We wish to consult and collaborate with health professions, fitness industry organisations and educational institutions to support the creation of a registered clinical exercise physiology professional, who can contribute to healthcare services.
- We wish to contribute to enhancing health outcomes and quality patient care pathways that lead to long-term exercise adherence.
- We wish to work with existing multidisciplinary teams of clinicians and allied health professionals across primary, secondary, tertiary and community-based services (public, private, charitable).
- We hope that this charter and roadmap will inspire healthcare leaders in other countries around the world to advocate for the inclusion of highly qualified exercise health professionals in healthcare systems.

## The problem

In the 21st century, urbanisation, automation and widespread use of the automobile have rapidly accelerated an underlying trend in sedentary behaviour.^1^ In the developed world, the impact of technological change on physical activity (PA) levels has occurred in line with increased numbers of individuals living longer. This raises the potential of individuals experiencing more years of physical and intellectual frailty and compromised life quality, with associated increases in healthcare costs.^2 3^ There has never been a more sedentary population of humans than the developed world in the 21st century, and recent evidence suggests that the positive historical trend in life expectancy may soon be compromised.^4 5^

Physical inactivity is a key factor in the development of non-communicable diseases^6^ and chronic non-communicable disease is correlated with, and exacerbated by, physical inactivity. In addition, low fitness levels are associated with increased morbidity and mortality.^6 7^ There is overwhelming evidence that regular exercise is vital for the successful management of chronic diseases.^8–10^ Indeed, for many conditions, exercise therapy may be as effective as pharmacological therapy.^10–12^ This is increasingly recognised in the UK. For example, the 2019 National Health Service (NHS) ‘Long Term Plan’ advocated exercise programmes for patients with cardiovascular disease (CVD) risk to prevent 14 000 premature deaths,^13^ and Public Health England acknowledged that embedding PA into clinical care pathways in acute settings is required.^14^

The UK has various public health strategies, largely supported by Public Health England and Sport England, to promote exercise and PA at a population level, including opportunities targeted at healthy individuals as well as some which focus on those known to be inactive or with known health inequalities (eg, for females ‘This Girl Can’ https://www.thisgirlcan.co.uk). These are self-directed pathways for those who choose to exercise, and they are delivered largely via the fitness industry, including public and private sector gyms and leisure centres. This approach has the benefits of low cost and broad accessibility to those who are generally young and/or apparently healthy. The approach for older individuals and/or those with chronic diseases (eg, Moving Healthcare Professionals https://www.sportengland.org/campaigns-and-our-work/moving-healthcare-professionals) involves health professionals (eg, primary and secondary care clinicians) advising individuals to begin or to do more PA/exercise.^15^ In primary care, general practitioners can also refer individuals with risk factors to an exercise referral scheme (ERS). ERS schemes were implemented UK-wide in the 1990s without evidence of robust effectiveness.^16^ Although there are some promising ERS examples (eg, in Wales,^17^ effectiveness^18^ and cost-effectiveness remains uncertain^19^); and patient uptake and adherence to exercise regimens is often poor.^20 21^ The ERS consists of prescription and delivery of an exercise programme (usually 12 weeks), largely focused on gym-based exercise prescription, by a fitness professional with vocational qualifications. Such vocational qualifications lack in-depth exercise science disciplinary knowledge, the clinical reasoning skills to prescribe personalised plans that include behaviour change^22^ and exercise testing and interpretation knowledge.^23^ There is accumulating evidence, outlined from meta-analyses, suggesting greater compliance to exercise regimens occur with supervision of exercise from a highly qualified exercise healthcare professional.^24 25^ Moreover, the clinical care pathways connecting ERS with secondary care exercise provision for cardiovascular, pulmonary and cancer rehabilitation are typically underdeveloped.^26^

Current programmes in the UK for the delivery of exercise for health benefit for individuals with established chronic disease occur in the ‘acute’ phase (eg, following an event or at diagnosis) and are typically aligned with highly specific populations (eg, exercise for cardiac rehabilitation or exercise for cancer pre/rehabilitation). For example, the British Association for Cardiovascular Prevention and Rehabilitation (BACPR) has been instrumental in standardising the delivery and supervision of exercise for cardiac services. The BACPR qualification is, however, only relevant to cardiac diseases and related comorbidities and does not fully transfer to other conditions such as cancer, respiratory, mental health, metabolic, kidney, musculoskeletal and neurological conditions; all of which have their own idiosyncratic issues in terms of exercise testing, prescription and supervision.^27–29^ There are a number of similar levels of qualification for other conditions also based on the National Occupational Standards at level 4 (eg, CanRehab, Later Life Training); but there is no collective qualification that encompasses multiple conditions. To highlight the example of cancer in the UK, a highly qualified exercise healthcare professional is urgently required, given emerging evidence that personalised exercise prescription on diagnosis can improve fitness prior to treatment and help alleviate the negative side effects of treatment including chemotherapy.^30–35^ This ‘prehabilitation’ service, which includes nutrition, psychological/behavioural change and exercise support, is being rolled out in the NHS across the UK, but there is currently no appropriately trained workforce to deliver the exercise component of this service.^36^

In summary, there is little standardisation of how exercise services are delivered across the UK, and by whom.^26^ Based on the evidence above, any services managing age-related and chronic diseases need a highly qualified exercise healthcare professional to be an integral part of the care pathway and team. Exercise testing, assessment, interpretation, prescription, delivery and outcomes evaluation for individuals with chronic and complex conditions requires a specialist knowledge base and expertise.^23^ Informed decision making and clinical reasoning for the optimisation of interventions and outcomes is best achieved by an exercise professional with advanced training in the prevention and management of chronic diseases including; cardiovascular, cancer, respiratory, metabolic, kidney, musculoskeletal, neurological and mental health. The problem in the UK is that there is currently no register, no accreditation system or regulator of clinical exercise physiologists within the health and social care system to ensure safe, effective and personalised exercise interventions for primary and secondary prevention, across the spectrum of chronic diseases.

## Do solutions exist?

A solution is to establish a ‘Clinical Exercise Physiologist’ in the UK able to work within care teams, across multiple chronic disease populations, where exercise interventions have an evidence base to show reduction in risk, improve quality of life and reduce healthcare costs. Such tertiary-qualified exercise professionals with formal registration and accreditation are already established in other countries around the world. This includes Certified Exercise Physiologists in the USA, Biokineticists in South Africa and Certified Clinical Exercise Physiologists in Canada. In the Australian public and private healthcare systems, accredited exercise physiologists (AEP) are uniquely recognised as allied health professionals with specialised education and training up to Masters level. AEPs specialise in the exercise testing, and the prescription and delivery of clinical exercise interventions for people with, or at high-risk of, chronic and complex medical conditions, injuries and disabilities. This Australian AEP model of qualification and professional recognition in the healthcare sector is widely regarded as the global gold-standard approach.

AEPs have a scope of practice that is based on foundational competency standards and evidence-based outcomes across a broad range of conditions.^37^ Core target pathologies for the AEP include, but are not limited to, cancer, cardiovascular, kidney, mental-illness, metabolic, musculoskeletal, neurological and neuromuscular, and respiratory/pulmonary diseases and conditions. AEPs conduct screening and assessments, and they apply independent clinical and scientific reasoning to develop safe and effective interventions that target the specific needs of individuals with chronic disease. Interventions delivered by AEPs are evidence based, and include specific exercise prescriptions, health and PA education, and support for lifestyle modification and PA behavioural change.

In Australia, there are currently ~6000 registered AEPs, working across healthcare systems including public and private hospitals, community-based primary care; occupational rehabilitation, veterans and aged health, and private clinics. Like other recognised services provided by allied health professionals, AEP exercise services are claimable under compensable funded schemes including those supported by the government (eg, Medicare, Veterans’ support, National Disability Insurance and WorkCover) and private health insurance. AEPs can work independently, but often work as part of multidisciplinary teams that include primary and secondary care physicians, physiotherapists and other allied health professionals.

Inclusion of AEPs in the Australian healthcare sector has conferred significant healthcare cost savings. Programmes involving AEPs that increase exercise are associated with annual well-being gains of US$7967 and US$11 847 per person with diabetes and CVD, respectively, with a benefit-cost ratio of 9:1 and 6:1.^38^ Compelling data also exist for the cost effectiveness of exercise in the treatment of mental health, dementia and other common chronic diseases.^38^ No other intervention possesses comparable impacts, across such a broad range of chronic diseases, as those associated with individualised exercise prescription delivered by AEPs.

Exercise physiology is one of several self-regulated allied health professions in Australia, and Exercise and Sports Science Australia (ESSA) is the approved legislated regulator and professional body. ESSA provides the scope of practice, professional and education standards. To gain accreditation, AEP’s must graduate from an accredited university programme that encompasses: (1) core exercise science standards, including biomechanics, exercise physiology, motor control and learning, exercise and sport psychology, and exercise prescription and delivery; (2) clinical exercise physiology standards, including professional practice, screening and assessment, and implementation of exercise and health interventions, as well as pathology-specific standards across a range of health domains and (3) 500 hours of supervised professional practical experience, including at least 360 hours in a clinical setting. Accredited university programmes are typically structured as 4-year undergraduate bachelor degrees or master programmes (equivalent to 1.5 years) that are completed following a Bachelor degree in exercise science. To remain accredited, which they need to do on a yearly basis, AEP’s are required to participate in ongoing annual professional development (points based system), ensure they have appropriate indemnity insurance (personally or through an employer), meet the recency of practice requirements, and have up to date cardiopulmonary resuscitation certificates if required in their current role^39 40^

As part of its regulatory role, university course accreditation is a core function of ESSA, which it delegates to the ESSA Accreditation Council. The Accreditation Council oversees accreditation policies and the appointment of course accreditation reviewers. Accreditation reviewers include experienced academics and accomplished practitioners, and they work in small teams of three to four members when carrying out a university programme review. Programmes may be accredited in exercise science (BSc), clinical exercise physiology (MSc) or both. In applying for accreditation, university programmes are required to map their curricula against the exercise science and clinical exercise physiology standards,^40^ and in doing so must demonstrate evidence of student attainment of each standard through learning and assessment activities. University programmes are also assessed on their level of resourcing, including the number and level of academic staff and their alignment with the fundamental domains of exercise science, support staff, facilities including laboratories and access to equipment. As the practicum programme forms an essential part of training and accreditation, university programmes are also assessed on their capacity to deliver supervised training opportunities that are aligned with the scope of practice across a broad range of professional placements. University programmes must demonstrate that the practicum programme is integrated into the programme curriculum, and that processes are in place to support placement supervisors and maintain the quality of the training and assessment activities undertaken. The university programme accreditation process is outlined in the accreditation guide.^41^

ESSA was founded in 1991 by a group of academics from prominent Australian Universities who recognised the potential of exercise to promote health. The academic community developed a roadmap that worked towards the common goal of securing recognition. Key elements were establishing (1) the scientific evidence base; (2) an accreditation pathway; (3) professional development and (4) continued education. The relevance and growth of exercise for health rapidly accelerated after recognition in 2005 by the Minister of Health in the Australian Federal Parliament that exercise specialists should play an important role in the public healthcare system of Australia (Medicare). This ‘Medicare Moment’ allowed trained and AEPs to join the allied health workforce and to bill Medicare for various services related to exercise testing and prescription in specific patient groups. Consequently, AEPs in Australia have become a vital professional ‘cog’ in the delivery of effective interventions to promote healthy ageing and as part of the prevention and management of chronic and complex diseases.

## Proposed regulation framework for clinical exercise physiologists in the UK

A key lesson learnt from ESSA and other international accrediting bodies is that for a clinical exercise physiologist to be a recognised and accredited allied health professional in the UK it is vital to establish an appropriate and effective system for the registration, regulation and education of practitioners. We propose to draw on the knowledge and experience of (1) the Australian AEP model, (2) other UK models that have realigned registration and accreditation for allied healthcare professionals (eg, Academy of Health Care Scientists, AHCS) and (3) the Department of Health and Social Care and Professional Standards Authority (PSA) frameworks for how registered titles (ie, Clinical Exercise Physiologist) can become a recognised and accredited profession.^42 43^ We propose three potential pathways that can be explored: (1) collaborate with an established regulator on the PSA register to facilitate accreditation and registration of ‘Clinical Exercise Physiology’ as a subtitle of a larger register^42^; (2) collaborate with an existing membership organisation/group of organisations to register a new ‘Clinical Exercise Physiology’ title and work together on the accreditation and registration processes or (3) establish a new stand-alone organisation, akin to ESSA in Australia, who would be responsible for registering a new ‘Clinical Exercise Physiology’ title and regulating controlling accreditation and registration. In any of these pathways, the following needs be completed:

Development of standards of competence and conduct that Clinical Exercise Physiology professionals must meet in order to be registered and practice. The development and implementation of professional standards should include standards of conduct, performance and ethics, including core clinical skills such as communication and record keeping.^43^ Standards of proficiency in Clinical Exercise Physiology, established in accordance with a standards development framework to ensure they are evidence based, benchmarked against international standards (eg, ESSA and Amercian College of Sports Medicine), and aligned with the clinical needs of patients and practitioners. On achieving the standards of proficiency, individuals would be eligible to apply for accreditation, and their application assessed by approved reviewers (trained and accredited to predefined standards). The content of application should closely match the professional standards and should allow ‘equivalence’ accreditation in the interim period before accredited courses produce their first graduates.

Assessment of the quality of education and training programmes to ensure they give Clinical Exercise Physiology graduates the skills and knowledge to practice safely and competently. Such standards of education and training will define the base level qualification for registration. Standards for University qualifications should include; programme admission requirements, programme governance, staffing and resources, curricula design and content, practice-based learning and assessment. A university programme accreditation framework should be developed based on current UK health service provision models with input on curriculum from the ESSA model. Accreditation review teams, comprised of experienced academics and practitioners, conduct site visits and assess university curricula and resources against the professional competency and education standards. Accreditation functions will include the approval of new programmes, the assessment of major programme changes, annual monitoring of programmes, and the management of complaints about programmes.

Development of standards of continuing professional development to ensure registered Clinical Exercise Physiology professionals continue to learn and develop and maintain their skills and knowledge so as to practise safely and effectively. Professional development standards are important to ensure the integrity and adherence of ongoing continuing professional development. It is essential that the ‘Clinical Exercise Physiology’ professional development framework mirrors and or is equivalent to other allied health professionals in the UK.

Establishment of a public, searchable database of registered Clinical Exercise Physiology professionals. Using the standards of proficiency a title of ‘Clinical Exercise Physiologist’ will fulfil the PSA requirements for a voluntary register. The organisation that registers the title will administer the register of Clinical Exercise Physiology professionals and will have oversight of all registration applications, including reregistration applications, international applications and appeals. This organisation will also maintain a public, searchable database of those registered as Clinical Exercise Physiologists.

Establishment of ‘Fitness to Practice’ processes to ensure registered Clinical Exercise Physiology professionals continue to meet the standards for training, professional skills, behaviour and health. Processes will be developed to manage allegations against registered Clinical Exercise Physiologists and allow for the collation of evidence to inform decisions on registrants’ Fitness to Practice.

## Road map to action

We have established an interim steering group (table 1) to provide leadership and momentum to bring together academics, health professionals, key stakeholders and organisations to work on the actions that will progress the registration of a ‘Clinical Exercise Physiologist’ title. We believe that, for ‘Clinical Exercise Physiologist’ to be a registered, recognised and accredited profession in the UK, collaboration and communication between stakeholders is an essential first step.

**Table 1:** Interim steering group established to provide leadership and momentum to bring together academics, health professionals, key stakeholders and organisations to work on the actions that will progress the registration of a ‘Clinical Exercise Physiologist’ title

Professor Helen Jones, Liverpool John Moores University	Winthrop Professor Daniel Green, The University of Western Australia
Professor Greg Whyte, Liverpool John Moores University, Board Member UK Active	Dr Christopher Askew, University of the Sunshine Coast, Australia Past President and Inaugural Chair of Professional Standards Council, Exercise and Sport Science Australia
Dr Keith Tolfrey, Loughborough University, Director International Confederation for Sport and Exercise Science Practice	Dr Andrew Scott, University of Portsmouth, Chair, British Association of Sport and Exercise Sciences (BASES) Clinical Exercise Science and Practice Interest Group
Professor Anna Campbell, Edinburgh Napier University, Director CanRehab	Professor Keith George, Liverpool John Moores University
Professor Helen Dawes, Oxford Brooks University, Director Centre for Movement and Occupational Rehabilitation Sciences	Professor Dawn Skelton, Glasgow Caledonian University, Director Later life Training, Co-Chair British Geriatrics Society Rehabilitation Group
Professor John Buckley, University Centre Shrewsbury, WHO Ischaemic Heart Disease and Rehabilitation 2030 Working Group Member	Dr Gordon McGregor, University Hospitals Coventry and Warwickshire NHS Trust and University of Coventry
Professor Sandy Jack, University of Southampton, Consultant Clinician Scientist, University Hospital Southampton NHS Foundation Trust	Professor David Broom, University of Coventry, BASES board member and Division of Physical Activity for Health, Chair
June Davis, Allied Health Professionals Advisor, Macmillian Cancer Support	

NHS, National Health Service.

We propose the interim steering group will engage with (1) stakeholders relevant to Sport and Exercise Science Graduates (~7500 per year), including, but not limited to, the Universities, the British Association for Sport and Exercise Sciences and the Physiological Society (Physoc); (2) organisations that currently provide training and vocational qualifications for clinical exercise provision including, but not limited to, BACPR, CanRehab, Later Life Training, Macmillan Cancer Support and low risk long-term conditions (CIMPSA); (3) the PSA and statutory regulators (eg, Health Care Professionals Council registered physiotherapists) or voluntary register holders (eg, AHCS); (4) NHS, other allied health professionals, medical societies and royal colleges (eg, Royal College of Anaesthetists), (5) other parties who express an interest in the agenda including other collaborative allied health professional (eg, Chartered Society of Physiotherapy) and medical organisations (eg, British Association of Sport and Exercise Medicine).

### In working towards the outlined framework the steering group will work on the following actions

1. Consultation: Approach other organisations on the PSA register for insight into the process and potential for combining resources.
2. Consultation: Explore how to engage with local and national government and NHS to advocate Clinical Exercise Physiologist role in the healthcare system including as placements for training and accreditation purposes.
3. Professional standards: Draft the standards of proficiency for consultation.
4. Professional standards: Engage other health professionals (eg, Sport and Exercise Medicine consultants, GPs, physiotherapist, commissioners) on the draft standards and clinical care pathway.
5. Education and accreditation: Bring together Universities to discuss potential degree course content and agree on an accreditation process and framework.
6. Regulatory body: Establish the regulator including register holder, accreditation body and professional development system and register the title.
7. Establish the council that leads and potentially governs the regulatory body that takes over from the interim steering group.

## Call to action

This charter presents a vision and a roadmap to establish, promote and regulate career paths for UK Clinical Exercise Physiologists.

We call on government and interested stakeholders to support the establishment of ‘Clinical Exercise Physiologists’ and engage with the interim steering group.

We call on university leaders and interested stakeholders to engage with the steering group and support the establishment of an independent body that can develop and administer an appropriate accreditation platform for UK Clinical Exercise Physiologists.

We call on the NHS and healthcare leaders to support this initiative by contributing to professional development, advocacy with politicians and other stakeholders and by employing appropriately accredited and regulated Clinical Exercise Physiologists.

We hope that this charter and roadmap will inspire healthcare leaders in other countries around the world to advocate for the inclusion of highly qualified exercise health professionals in healthcare systems.

## Data Availability

Data sharing not applicable as no datasets generated and/or analysed for this study. No data are available. No data used in the document.

## References

[1] Shephard RJ. A short history of occupational fitness and health promotion. Prev Med 1991;20:436–45. 10.1016/0091-7435(91)90041-2 1862064

[2] Church TS, Thomas DM, Tudor-Locke C, et al. Trends over 5 decades in U.S. occupation-related physical activity and their associations with obesity. PLoS One 2011;6:e19657. 10.1371/journal.pone.0019657 21647427 PMC3102055

[3] Odden MC, Coxson PG, Moran A, et al. The impact of the aging population on coronary heart disease in the United States. Am J Med 2011;124:827–33. 10.1016/j.amjmed.2011.04.010 21722862 PMC3159777

[4] Curtin SC. Trends in cancer and heart disease death rates among adults aged 45-64: United States, 1999-2017. Natl Vital Stat Rep 2019;68:1–8. 32501204

[5] Olshansky SJ. Projecting the future of U.S. health and longevity. Health Aff 2005;24:W5-R86–W5-R89. 10.1377/hlthaff.W5.R86 16186155

[6] Lee I-M, Shiroma EJ, Lobelo F, et al. Effect of physical inactivity on major non-communicable diseases worldwide: an analysis of burden of disease and life expectancy. Lancet 2012;380:219–29. 10.1016/S0140-6736(12)61031-9 22818936 PMC3645500

[7] Blair SN. Physical inactivity: the biggest public health problem of the 21st century. Br J Sports Med 2009;43:1–2. 19136507

[8] Blair SN, Sallis RE, Hutber A, et al. Exercise therapy - the public health message. Scand J Med Sci Sports 2012;22:e24–8. 10.1111/j.1600-0838.2012.01462.x 22429265

[9] Fiuza-Luces C, Garatachea N, Berger NA, et al. Exercise is the real polypill. Physiology 2013;28:330–58. 10.1152/physiol.00019.2013 23997192

[10] Pedersen BK, Saltin B. Exercise as medicine - evidence for prescribing exercise as therapy in 26 different chronic diseases. Scand J Med Sci Sports 2015;25 Suppl 3:1–72. 10.1111/sms.12581 26606383

[11] Futenma K, Asaoka S, Takaesu Y, et al. Impact of hypnotics use on daytime function and factors associated with usage by female shift work nurses. Sleep Med 2015;16:604–11. 10.1016/j.sleep.2014.11.018 25890782

[12] Walsh J et al. Effects of exercise training on conduit and resistance vessel function in treated and untreated hypercholesterolaemic subjects. Eur Heart J 2003;24:1681–9. 10.1016/S0195-668X(03)00384-1 14499232

[13] NHS. The NHS long term plan. NHS, 2019.

[14] England PH, Active E. Every day: an evidence-based approach to physical activity. London: Pulblic Health England, 2014.

[15] Brannan M, Bernardotto M, Clarke N, et al. Moving healthcare professionals - a whole system approach to embed physical activity in clinical practice. BMC Med Educ 2019;19:84. 10.1186/s12909-019-1517-y 30876426 PMC6419815

[16] Sowden SL, Raine R. Running along parallel lines: how political reality impedes the evaluation of public health interventions. A case study of exercise referral schemes in England. J Epidemiol Community Health 2008;62:835–41. 10.1136/jech.2007.069781 18701737

[17] Murphy SM, Edwards RT, Williams N, et al. An evaluation of the effectiveness and cost effectiveness of the National exercise referral scheme in Wales, UK: a randomised controlled trial of a public health policy initiative. J Epidemiol Community Health 2012;66:745–53. 10.1136/jech-2011-200689 22577180 PMC3402741

[18] Williams NH, Hendry M, France B, et al. Effectiveness of exercise-referral schemes to promote physical activity in adults: systematic review. Br J Gen Pract 2007;57:979–86. 10.3399/096016407782604866 18252074 PMC2084138

[19] Campbell F, Holmes M, Everson-Hock E, et al. A systematic review and economic evaluation of exercise referral schemes in primary care: a short report. Health Technol Assess 2015;19:1–110. 10.3310/hta19600 PMC4781341 26222987

[20] Pavey TG, Anokye N, Taylor AH, et al. The clinical effectiveness and cost-effectiveness of exercise referral schemes: a systematic review and economic evaluation. Health Technol Assess 2011;15:i. 10.3310/hta15440 PMC4781450 22182828

[21] Pavey T, Taylor A, Hillsdon M, et al. Levels and predictors of exercise referral scheme uptake and adherence: a systematic review. J Epidemiol Community Health 2012;66:737–44. 10.1136/jech-2011-200354 22493474

[22] Buckley BJR, Thijssen DHJ, Murphy RC, et al. Making a move in exercise referral: co-development of a physical activity referral scheme. J Public Health 2018;40:e586–93. 10.1093/pubmed/fdy072 29688551

[23] Franklin B, Fern A, Fowler A, et al. Exercise physiologist's role in clinical practice. Br J Sports Med 2009;43:93–8. 10.1136/bjsm.2008.055202 19050005

[24] Vancampfort D, Rosenbaum S, Schuch FB, et al. Prevalence and predictors of treatment dropout from physical activity interventions in schizophrenia: a meta-analysis. Gen Hosp Psychiatry 2016;39:15–23. 10.1016/j.genhosppsych.2015.11.008 26719106

[25] Stubbs B, Vancampfort D, Rosenbaum S, et al. Dropout from exercise randomized controlled trials among people with depression: a meta-analysis and meta regression. J Affect Disord 2016;190:457–66. 10.1016/j.jad.2015.10.019 26551405

[26] Crozier A, Watson PWG, George LE, et al. Clinical exercise provision in the UK: a mapping study. BMJ open SEM. In Press.

[27] Scharhag-Rosenberger F, Kuehl R, Klassen O, et al. Exercise training intensity prescription in breast cancer survivors: validity of current practice and specific recommendations. J Cancer Surviv 2015;9:612–9. 10.1007/s11764-015-0437-z 25711667

[28] Thompson PD, Arena R, Riebe D, et al. ACSM's new preparticipation health screening recommendations from ACSM's guidelines for exercise testing and prescription, ninth edition. Curr Sports Med Rep 2013;12:215–7. 10.1249/JSR.0b013e31829a68cf 23851406

[29] Colberg SR, Sigal RJ, Fernhall B, et al. Exercise and type 2 diabetes: the American College of sports medicine and the American diabetes association: joint position statement. Diabetes Care 2010;33:e147–67. 10.2337/dc10-9990 21115758 PMC2992225

[30] van Rooijen S, Carli F, Dalton S, et al. Multimodal prehabilitation in colorectal cancer patients to improve functional capacity and reduce postoperative complications: the first international randomized controlled trial for multimodal prehabilitation. BMC Cancer 2019;19:98. 10.1186/s12885-018-5232-6 30670009 PMC6341758

[31] West MA, Astin R, Moyses HE, et al. Exercise prehabilitation may lead to augmented tumor regression following neoadjuvant chemoradiotherapy in locally advanced rectal cancer. Acta Oncol 2019;58:588–95. 10.1080/0284186X.2019.1566775 30724668

[32] Borch KB, Braaten T, Lund E, et al. Physical activity before and after breast cancer diagnosis and survival - the Norwegian women and cancer cohort study. BMC Cancer 2015;15:967. 10.1186/s12885-015-1971-9 26672980 PMC4682279

[33] Holmes MD, Chen WY, Feskanich D, et al. Physical activity and survival after breast cancer diagnosis. JAMA 2005;293:2479–86. 10.1001/jama.293.20.2479 15914748

[34] Schmitz KH, Campbell AM, Stuiver MM, et al. Exercise is medicine in oncology: engaging clinicians to help patients move through cancer. CA Cancer J Clin 2019;69:468–84. 10.3322/caac.21579 31617590 PMC7896280

[35] Meyerhardt JA, Giovannucci EL, Holmes MD, et al. Physical activity and survival after colorectal cancer diagnosis. J Clin Oncol 2006;24:3527–34. 10.1200/JCO.2006.06.0855 16822844

[36] Bates A, West MA, Jack S. Framework for prehabilitation services. Br J Surg 2020;107:e11–14. 10.1002/bjs.11426 31903594

[37] ESSA. Accredited exercise physiologist scope of practice. ESSA, 2018.

[38] Deloitte. Value of accredited exercise Physiologists in Australia. Deloitte, 2015.

[39] ESSA. Exercise science standards. Queensland: ESSA, 2013.

[40] ESSA. Accredited exercise physiologist professional standards. ESSA, 2015.

[41] ESSA. Course accreditation guide: for education providers. ESSA, 2019.

[42] PSA. Regulation rethought: proposals for reform. London: PSA, 2016.

[43] PSA. Standards for accredited registers. London: PSA, 2016.

